# A Combined Approach Using Patch-Clamp Study and Computer Simulation Study for Understanding Long QT Syndrome and TdP in Women

**DOI:** 10.2174/157340308786349507

**Published:** 2008-11

**Authors:** Tetsushi Furukawa, Junko Kurokawa, Colleen E Clancy

**Affiliations:** 1 Department of Bio-Informational Pharmacology, Madical Research Institute, Tokyo Medical and Dental University; 2 Department of Physiology and Biophysics, Institute for Computational Biomedicine, Weill Medical College of Cornell University

**Keywords:** Long QT syndrome, sex hormone, nitric oxide, arrhythmia, patch-clamp, non-genomic pathway

## Abstract

Female sex is an independent risk factor for development of torsade de pointes (TdP)-type arrhythmias in both congenital and acquired long QT syndrome (LQTS). In females, QT_c_ interval and TdP risk vary during the menstrual cycle and around delivery. Biological experiments including single-cell current recordings with the patch-clamp technique and biochemical experiments show that progesterone modulates cardiac K^+^ current and Ca^2+^ current *via* the non-genomic pathway of the progesterone receptor, and thus the cardiac repolarization duration, in a concentration-dependent manner. Incorporation of these biological findings into a computer model of single-cell and coupled-cell cardiomyocytes simulates fluctuations in QT_c_ interval during the menstrual cycle with reasonable accuracy. Based on this model, progesterone is predicted to have protective effects against sympathetic nervous system-induced arrhythmias in congenital LQTS and drug-induced TdP in acquired LQTS. A combined biological and computational approach may provide a powerful means to risk stratify TdP risk in women.

## INTRODUCTION

A growing body of evidence suggests that clinical arrhythmia syndromes emerge as a result of complicated interactions of multiple endogenous and environmental factors. A combined approach using patch-clamp study and computer simulation study is a powerful means for investigating the influence of multiple interacting factors on the development of clinical symptoms. In this mini-review, we will discuss our recent work using a combined biological and computational approach to predict arrhythmic risks in women.

## 1. ARRHYTHMIAS IN LONG QT SYNDROME (LQTS) IN WOMEN

LQTS is a cardiac arrhythmia syndrome characterized by prolonged QT intervals on the 12-lead surface electrocardiogram, polymorphic ventricular tachyarrhythmias with unique morphology, called *torsade de pointes* (TdP), and syncope and sudden death. Experiments using multicellular wedge prepatnion indicate that TdP is triggered by early afterdepolarization (EAD) followed by intramural phase 2 reentry, which is based on hetelogeneous prolongation myocardial action potential duration (APD) [3]. APD prolongation is caused by either suppression of outward currents including transient outward current (I_to_), and rapidly-activating and slowly-activating delayed rectifier K^+^ current (I_Ks_ and I_Kr_), or/and enhancement of inward currents including L-type Ca^2+^ current (I_Ca,L_) and persistent Na^+^ current (I_Na_).

LQTS occurs as a congenital form or an acquired form. In both congenital and acquired LQTS, female sex is an independent risk factor for the development of TdP [1, 2]. In females, there are dynamic fluctuations in QT_c_ interval and the risk of TdP during the menstrual cycle [4]. Although several previous studies did not find QT_c_ interval differences among the different menstrual phases [5, 6], a recent study analyzing various parameters of cardiac repolarization finds that repolarization duration is shorter in the luteal phase than in the follicular phase by about 10 msec [6]. Ibutilide is a class III antiarrhythmic agent that prolongs QT_c_ interval in a dose-dependent manner, and is used for termination of atrial fibrillation and atrial flutter. QT_c_ prolongation induced by ibutilide is the greatest during menses (63 msec), intermediate in ovulation (59 msec), and the least in the luteal phase (53 msec) [5]. In these studies [5, 7], serum sex hormone level was determined: serum progesterone level was higher in the luteal phase than in the follicular phase, during menses, and in ovulation, while serum 17β-estradiol level was not significantly different between the luteal phase and the follicular phase. Thus, progesterone is suggested to be responsible for differences in cardiac repolarization duration and in ibutilide-induced QT_c_ prolongation during the menstrual cycle.

In post-menopausal women, although earlier studies report conflicting data for effects of hormone replacement therapy on QT_c_ interval [8-10]. a recent study consisting of a large study population indicates that hormone replacement therapy with estrogen alone causes slight but significant prolongation of QT_c_ interval by about 2 msec, while combinational hormone replacement therapy with estrogen and progestin consistently shortens QT_c_ interval by about 1 msec [11]. Effects of pregnancy in LQTS patients were also examined [12, 13]. In careful survey of arrhythmia events in congenital LQTS patients around delivery, new-onset of arrhythmia events increased postpartum where progesterone level falls dramatically compared to before or during pregnancy [12]. Taken together, the luteal hormone, progesterone, is strongly suggested to have protective effects against long QT-associated arrhythmias.

## 2. GENOMIC EFFECTS OF PROGESTERONE ON CARDIAC ION CHANNELS

**Fig (1). Genomic and non-genomic pathway of sex hormones.**
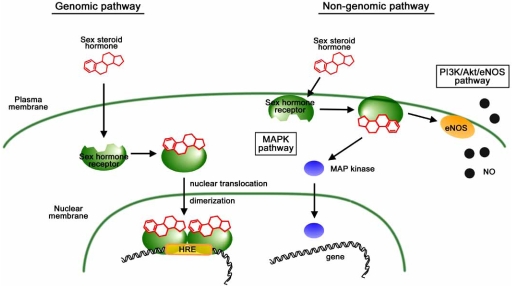
In the genomic pathway, sex steroid hormones penetrate into cells, and bind to receptors in the cytosol. The ligand/receptor complex then translocates into the nucleus, binds to the genes with hormone responsive element (HRE), and regulates gene expression. In the non-genomic pathway, sex hormones release nitric oxide *via* the PI3-kinase/Akt/eNOS pathway or activate MAP-kinase in a membrane-delimited manner.

Progesterone belongs to lipophilic gonadal steroid hormone family, whose canonical pathway is to permeate into cell across surface membrane, binds to intracellular receptor, translocates into the nucleus as a ligand/receptor complex form, and binds to a gene containing a hormone responsive element (Fig. **1**) [14-16]. In addition to this “genomic action”, for the last decade sex hormones have been shown to exhibit rapid actions which cannot be explained by genomic action and are referred to as “non-genomic action” (Fig. **1**) [17-20]. Non-genomic action takes place in a membrane-delimited manner: PI3-kinase/Akt-dependent activation of endothelial nitric oxide synthase (eNOS) [21, 22] and activation of MAP-kinase [23, 24] are the two most well characterized signaling pathways.

Previous studies of effects of progesterone on cardiac ion channels have mostly dealt with its genomic actions. Song *et al*. [25] examined effects of gonadal steroids on expression of transient outward current channels, K_V_4.3, using a myometrium heterologous expression as a model system. They found that 4 days-injection of 17β-estradiol (50 μg/ml) decreased expression of K_V_4.3, whereas injection of progesterone (3 mg/ml) did not affect K_V_4.3 expression. The α1C subunit of the L-type Ca^2+^ current (I_Ca,L_) channel can be detected as a 240 kDa long form (α1C long) and a 190 kDa short form (α1C short). In myometrium, 17β-estradiol decreased the long α1C form/short α1C form (L/S ratio), while progesterone increased the L/S ratio; in brain or heart, either 17β-estradiol or progesterone did not change the L/S ratio [26]. Thus, the genomic effects of progesterone on cardiac repolarization are currently undefined and cannot explain a protective effect of progesterone against TdP risk.

## 3. NON-GENOMIC EFFECTS OF PROGESTERONE ON CARDIAC ION CURRENTS

Major currents determining cardiac repolarizaion are I_Ks_, I_Kr_, and I_Ca,L_ in human and guinea-pig. I_to_ and I_Ca,L_ are critical in mouse and rat [27, 28]. Thus, we used cardiac myocytes isolated from guinea pig left ventricle to investigate acute effects of progesterone. Sympathetic nervous system (SNS) stimulation is a critical triggering factor for TdP in LQTS patients [29], and thus we examined both the basal condition and the SNS stimulation-mimicked condition with isoproterenol application or with intracellular dialysis of cAMP and okadaic acid (OA). Progesterone at a concentration of 100 nM shortened APD both in the basal condition and the SNS-stimulated condition.^29^ Progesterone-induced APD shortening is *via* the non-genomic pathway, since progesterone-induced APD shortening was observed within a few minutes, reached steady-state within 10 min, and was inhibited by a specific progesterone receptor inhibitor, mifepristone (1 μM).

**Fig (2). Two distinct mechanisms for NO actions.**
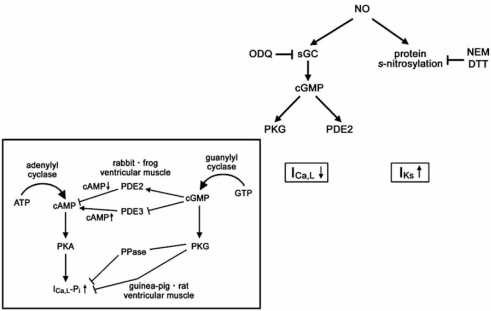
NO activates sGC, and produced cGMP regulates PKG, PDE2, PDE3, and/or protein phosphatase (PPase). NO also induces protein *s*-nitrosylation. I_Ca,L_ suppression by progesterone is *via* the sGC/cGMP pathway, while I_Ks_ activation is *via* the protein *s*-nitrosylation. Inset shows the antagonistic action between cAMP and cGMP for I_Ca,L_ regulation. It has been reported that in rabbit and frog ventricular myocytes, it occurs at the PDE2 level, while in guinea-pig and rat ventricular myocytes, it occurs at the PKG level [34]. The inhibition of PDE3 by cGMP and the activation of PPase by cGMP/PKG signaling pathway may also be involved.

The ionic mechanism underlying APD shortening by progesterone is to modulate I_Ks_ and I_Ca,L_, but not I_Kr_. In the basal condition, progesterone enhanced I_Ks_ in a concentration-dependent manner with an EC_50_ value of 2.7 nM, while progesterone did not significantly affect I_Ca,L_ (Fig. **2A**) [30]. SNS-stimulation caused enhancement of both I_Ca,L_ and I_Ks_. Further application of progesterone reduced I_Ca,L_ to the level before cAMP and OA application, while it did not significantly change I_Ks_ [30]. The IC_50_ value for I_Ca,L_ suppression was 29.9 nM (Fig. **2B**).

The biophysical mechanism for regulation of I_Ks_ and I_Ca,L_ is different. The effects of progesterone on I_Ks_ were frequency- and voltage-independent [30]. In contrast, progesterone caused a positive shift in the I_Ca,L_ activation curve and a negative shift in the inactivation curve [30]. Computer simulation analysis showed that changes in current conductance without changes in current kinetics reproduced the effects of progesterone on I_Ks_ observed in biological experiments. Changes in voltage dependency alone with no change in current conductance reproduced the effects of progesterone on I_Ca,L_ with a high accuracy. Thus, effects of progesterone on I_Ks_ are mainly to alter current conductance and modulate I_Ca,L_ by affecting current kinetics.

**Fig (3). Non-genomic regulation of IKs and ICa,L by progesterone in patch-clamp experiments.**
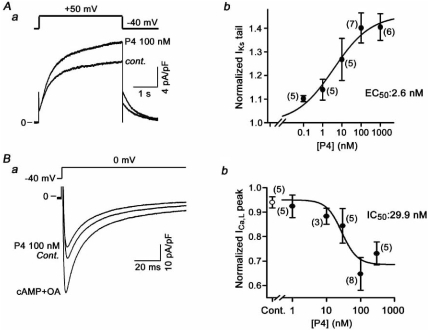
**A**) In the basal condition, progesterone (P4) enhances I_Ks_. ***a,*** Representative superimposed I_Ks_ current traces in the control and after treatment with 100 nM P4. I_Ks_ was elicited by depolarization to +50 mV from a holding potential of -40 mV at 0.1 Hz. ***b***, Concentration-response curve for I_Ks_ enhancement by progesterone. EC_50_ was 2.6 nM. **B**) In the SNS-stimulation-mimicked condition, progesterone suppressed IC_a,L_. ***a,*** Representative superimposed I_Ca,L_ current traces in the control, during intracellular dialysis of cAMP and OA, and after treatment with 100 nM P4 in the continued presence of cAMP and OA. I_Ca,L_ was elicited by depolarization from a holding potential of -40 mV to 0 mV at 0.1 Hz. ***b,*** Concentration-response curve for I_Ca,L_ suppression by progesterone. IC_50_ was 29.9 nM.

Despite distinct biophysical mechanism for I_Ks_ and I_Ca,L_ regulation, the principal mediator for both I_Ks_ enhancement in the basal condition and I_Ca,L_ suppression in the SNS-stimulated condition appears to be nitric oxide (NO), since both were abolished by nitric oxide (NO) trappers and eNOS inhibitors [31, 32]. However, the mechanism by which NO modulates I_Ks_ and I_Ca,L_ appears to be different. I_Ca,L_ suppression by progesterone was abolished by an inhibitor of soluble guanylyl cyclase (sGC), indicating that I_Ca,L_ is regulated by progesterone *via* a NO/sGC/cGMP axis (Fig. **3**) [33]. Antagonistic action of cAMP and cGMP for I_Ca,L_ has been demonstrated, which appears to vary among species [34]. In rabbit and frog ventricular myocytes, cGMP antagonizes cAMP effects by promoting cAMP breakdown by activating cGMP-dependent phosphodiesterase (PDE2) [34]. In guinea-pig and rat ventricular myocytes, cAMP-dependent protein kinase (PKA) phosphorylates the α-subunit of I_Ca,L_ and enhances I_Ca,L_ only in the presence of A-kinase anchoring protein (AKAP) [34]. cGMP-dependent protein kinase (PKG) phosphorylates both the α-subunit and the β-subunit of I_Ca,L_ [35]. Phosphorylation of the α-subunit by PKG does not affect I_Ca,L_, likely due to the absence of AKAP, while phosphorylation of the β-subunit antagonizes the effect of the α-subunit phosphorylation by PKA [35]. In addition, the inhibition of PDE3 by cGMP to enhance the cAMP-induced activation and facilitation of I_Ca,L_, and activation of protein phosphatase *via* cGMP-PKG signaling pathway to suppress the cAMP-mediated facilitation may contribute to the complicated interaction of cAMP and cGMP in the heart.

On the other hand, I_Ks_ enhancement was not inhibited by a sGC inhibitor, but was inhibited by a thiol-alkylating reagent, *N*-ethylmaleimide, and a reducing reagent, di-thiothreitol [33]. These data suggest that cGMP-independent mechanisms, possibly protein *s*-nitrosylation, play a role for I_Ks_ enhancement (Fig. **3**) [33]. Protein *s*-nitrosylation is the direct NO transfer to the thiol residue of Cys, is highlighted as a novel mechanism of protein post-translational modification [36, 37], and occurs independent of cAMP. Thus, it is possible that progesterone regulates I_Ks_ in the basal condition and I_Ca,L_ only in the SNS-stimulated condition. However, it remains to be proven if the I_Ks_ channel is indeed *s*-nitrosylated. If that is the case, it is also undetermined whether the α-subunit, KCNQ1, or the β-subunit, KCNE1, is the target of s-nitrosylation, what is the underlying mechanism for specific *s*-nitrosylation of KCNQ1 or KCNE1, and how s-nitrosylation induces I_Ks_ channel activation. 

## 4. COMPUTATIONAL SIMULATION OF THE EFFECTS OF PROGESTERONE

QT_c_ interval and TdP risk are regulated by various factors, including SNS status, heart rate, medications, serum electrolyte level, and others. Our biological experiments suggest progesterone as an additional major factor that modulates QT_c_ interval and TdP risk. Since progesterone level varies during the menstrual cycle and around delivery, progesterone effects may contribute to the fluctuation of QT_c_ interval and TdP risk during the menstrual cycle and pregnancy. Since a computational approach is especially powerful to simulate these changes, our first challenge was to investigate if incorporating effects of progesterone in the cardiac APD computer model reproduces fluctuation of APD during the menstrual cycle. 

**Fig (4). The effects of progesterone on simulated cardiac action potentials.**
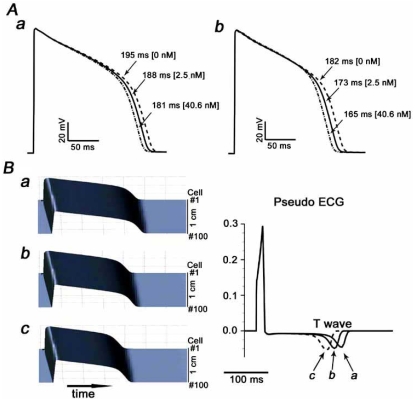
**A**) Simulated action potentials in the absence (***a***) and presence (***b***) of simulated SNS stimulation under baseline conditions (dashed line) and with 2.5 nM (solid line) and 40.6 nM (dashed-dot-dot line) progesterone. The 10th paced beat at a pacing interval of 400 ms is shown. **B**) Left panel: Simulated action potentials (10th paced beat at a 400 ms pacing interval) in a 1 cm cardiac fiber (cell number=100; top is cell #1, and bottom is cell #100). An action potential was elicited at cell #1, and propagated from top to bottom. ***a***, Baseline (no SNS stimulation and no progesterone). ***b***, With 40.6 nM progesterone. ***c***, With 40.6 nM progesterone in the presence of SNS stimulation. Right panel: Computed virtual electrograms under the three conditions. The corresponding T-waves are indicated with arrows.

We incorporated effects of progesterone obtained in our biological experiments in the Faber-Rudy model of the guinea pig myocyte [38]. Since reported progesterone level in women is ~2.5 nM in the follicular phase and ~40.6 nM in the luteal phase [39], we incorporated effects of progesterone at 2.5 nM and at 40.6 nM. The model predicts that progesterone at 40.6 nM shortens APD by 3.7 % under basal conditions and 4.6 % under SNS-stimulated conditions compared to APD at 2.5 nM progesterone (Fig. **4**) [30]. Clinically observed QT intervals are shorter by about 2.4%-2.8 % in the luteal phase than in follicular phase [5], and so the APD shortening predicted in the model (3.7-4.6 %) fits well with the observed fluctuation in QT interval during the menstrual cycle in women.

Effects of progesterone in a single cell do not necessarily predict the effect at the multi-cell level, organ level, or *in vivo* level. As a first step to simulate effects of progesterone in higher dimensions, we constructed a coupled-cells model, in which 100 cardiomyocytes are electrotonically connected with simulated resistances between them to represent gap-junctions. We then investigated the effects of progesterone and SNS in simulated coupled tissue and computed virtual electrograms from simulated gradients of depolarization and repolarization. Simulations suggest that during the luteal phase when progesterone = 40.6 nM, maximal SNS may additionally shorten QT interval by 12.2 % (Fig. **4**) [30]. These simulations support the notion that progesterone may exert protective QT shortening effects under conditions on SNS.

## 5. PREDICTED EFFECTS OF PROGESTERONE AGAINST ARRHYTHMIA

**Fig (5). Progesterone may protect against long QT-related arrhythmia.**
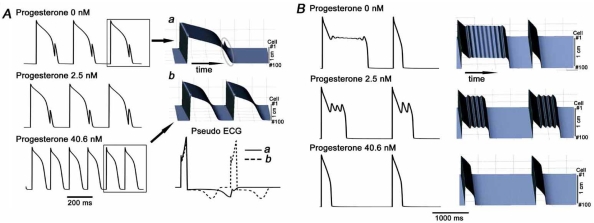
**A**) The effects of progesterone on arrhythmic rhythms in congenital LQTS. Progesterone improves action potential adaptation in congenital LQTS (LQTS5) at fast heart rates during SNS stimulation. We simulated the D76N mutation in the I_Ks_ β-subunit KCNE1 that disrupts regulation of I_Ks_ by protein kinase A. Left panel: Shown are 6 action potentials (15th - 20th) elicited from cells with D76N I_Ks_ at a fast rate (CL = 150 ms) during the SNS stimulation in the absence (top panel), and in the presence of 2.5 nM (middle panel) or 40.6 nM (bottom panel) progesterone. Right Panel: Simulated propagation of action potentials in paced (150 ms, 29th and 30th beats are shown) one-dimensional tissue in the absence of progesterone (***a***), in the presence of 40.6 nM progesterone (***b***) and the corresponding computed electrogram. A gray circle in panel ***a*** highlights failure of propagation of the second stimulus, which is applied during the mutation induced extended refractory period. **B**) The effects of progesterone on EADs resulting from acquired LQTS simulated by IKr block. Traces of the 9th and 10th action potentials during 50% IKr block in the absence (top panel), and in the presence of 2.5 nM (middle panel) or 40.6 nM (bottom panel) progesterone. The cycle length is 2000 ms. The left panel shows the single cells and the right panel shows results in corresponding fibers under the same conditions.

Since the model reproduces the effects of progesterone on APD in patch-clamp experiments and QT_c_ variation during the menstrual cycle in women with a good accuracy, our next step was to utilize this model to predict the effects of progesterone on LQTS-associated arrhythmia susceptibility. To examine the effects on SNS-induced arrhythmias, we used the D76N KCNE1 mutation linked to congenital LQTS5. I_Ks_ exhibits accumulation in the pre-open state during the rapid heart rates, resulting in action potential adaptation [40]. SNS stimulation enhances I_Ca,L_ to increase Ca^2+^ influx [41]. SNS stimulation also enhances I_Ks_ [42, 43] that counter-balances I_Ca,L_ enhancement and maintains APD within a certain range [44]. In LQTS1 and LQTS5, I_Ks_ channel disturbance results in dysfunction of action potential adaptation to rapid heart rates and response to SNS stimulation. The D76N KCNE1 mutation reduces the current and renders the I_Ks_ channel insensitive to β-adrenergic stimulation [45], thus probands carrying D76N KCNE1 mutation readily develop TdP with SNS stimulation at rapid heart rates [46]. In the absence of progesterone, the mutant model cells are unable to adapt to the fast pacing frequency because I_Ks_ fails to increase in response to the SNS stimulation (Fig. **5A**) [30]. Interestingly, both in the single-cell and coupled-cell model in the presence of progesterone at 2.5 nM, some improvement is observed; in the presence of 40.6 nM, a failed SNS stimulation response is compensated for by the action of progesterone alone to increase I_Ks_ (Fig. **5A**) [30]. Thus, enhancement of I_Ks_ in the absence of SNS stimulation, and inhibition of cAMP-induced I_Ca,L_ by progesterone improve action potential adaptation, which is dependent on progesterone level. These simulations suggest a mechanism for SNS-related arrhythmic risk varies during the menstrual cycle in women.

Drug-induced TdP is believed to occur by blockade of the *human ether-a-go-go related gene* (*hERG*) channel by drugs with various structures [47], and at slow heart rates. In a simulation, severe EADs were induced by 50% block of I_Kr_ at a slow heart rate (30 bpm) (Fig. **5B**). At 2.5 nM of progesterone, some improvement is observed (middle panel); at 40.6 nM of progesterone, the EADs are abolished and the action potential morphology is normalized (Fig. **5B**). Thus, progesterone is predicted to have protective effects against drug-induced arrhythmias, which also fluctuate during the menstrual cycle. Progesterone does not have apparent effects on I_Kr_ (data not shown), and thus predicted protection against drug-induced EAD may be attributed to an increase in repolarization reserve by I_Ks_ enhancement [48].

## CONCLUSION

Our patch-clamp experiment demonstrates that the non-genomic effect of the sex hormone progesterone constitutes a novel regulatory mechanism of cardiac repolarization. Serum progesterone level fluctuates during the menstrual cycle: within this level, progesterone modulates I_Ks_ and I_Ca,L_ and, therefore, is partly responsible for the cyclic changes in QT_c_ interval and TdP risk during the menstrual cycle. A computational approach allows for simulation of multi-factorial and periodical phenomenon. Incorporation of progesterone effects observed in our biological study into the computational model reproduces cyclic changes in QT_c_ interval, and predicts dose-dependent protective effects of progesterone against SNS-stimulation-induced and drug-induced arrhythmias. This approach provides a first step to risk stratify TdP arrhythmias in women. To improve this approach, further efforts are certainly needed, which include the elucidation of; (1) the effects of sex hormones other than progesterone, including various estrogen metabolites; (2) genomic effects of progesterone and estrogens; and (3) simulation at the organ and *in vivo* level.
